# An overview of paediatric autoimmune and genetic cholestatic liver disease for the adult physician

**DOI:** 10.1016/j.clinme.2025.100533

**Published:** 2025-11-19

**Authors:** Vikram Bains, Deepak Joshi

**Affiliations:** Institute of Liver Studies, King’s College Hospital NHS Foundation Trust, London, UK

**Keywords:** Autoimmune hepatitis, Cholestasis, Paediatric disorders, Genetic, Liver transplant

## Abstract

Paediatric chronic liver diseases are rare, but are increasingly encountered in adult practice as survival improves and more adolescents transition to adult services. Adult physicians must be familiar with these conditions, recognise complications and implement high-quality management of these conditions to provide safe, effective care. Autoimmune liver disease in children includes autoimmune hepatitis, autoimmune sclerosing cholangitis and primary sclerosing cholangitis, with distinct serological and clinical profiles. Cholestatic disorders such as progressive familial intrahepatic cholestasis and Alagille syndrome present additional challenges, often impacting multiple organs and requiring multidisciplinary care. Many patients will enter adulthood having undergone liver transplant, requiring long-term immunosuppression and, where relevant, family planning advice. Recognition of extra-hepatic manifestations, metabolic complications and mental health issues is essential to holistic management. This article outlines the key paediatric liver diseases relevant to adult practice, highlighting key elements of care and long-term considerations for this unique and often complex patient population.

## Highlights

• Outcomes of paediatric liver disease are improving which is resulting in increasing numbers of patients transitioning to adult services.
• Autoimmune liver disease can be associated with other autoimmune conditions and optimising care in adult services to prevent complications is crucial.
• Autoimmune sclerosing cholangitis can progress to a primary sclerosing phenotype in adulthood.
• Cholestatic disorders are diverse and can present with different phenotypes in children and adults.
• Young adults often require multi-disciplinary input to provide holistic care relating to conditions and complications associated with their liver disease. This can include family planning.

## Introduction

Paediatric chronic liver disease is rare, but understanding and management of this group of disorders has improved, leading to increased numbers of adolescents transitioning to adult liver services.^1^ The combination of these rare disorders, with the often-complex needs of this young population, can present unique challenges to adult services across many specialties. Adult physicians require awareness and knowledge of these diseases and complications to ensure that they provide ongoing high-quality care. Paediatric liver disease encompasses a broad range of conditions, but this article focuses on autoimmune liver disease, the three most common types of progressive familial intrahepatic cholestasis (PFIC) and Alagille syndrome.

## Transitioning to adult services

Young adults (YA) with complex medical conditions must often balance several components linked to their conditions. These include the impact on their education and finances, as well as the impact of ill health on their relationships and family planning.^1^ Mental health disorders are also crucial to detect and manage in this cohort as they can impair health outcomes through medication non-adherence, for example.^1^ Holistic management of YAs requires an individualised multidisciplinary approach to facilitate ongoing effective care in adult services. Psychological and social support services form key components of this approach. Guidelines are available which highlight the key components of a transition service.^1^,^2^

## Paediatric liver diseases

### Autoimmune liver disease

Paediatric autoimmune liver disease (AILD) encompasses autoimmune hepatitis (AIH), autoimmune sclerosing cholangitis (ASC) and primary sclerosing cholangitis (PSC) with reported incidence rates of 0.4, 0.1 and 0.2 cases/100,000 children respectively.^3^

AIH is a chronic, inflammatory condition that can lead to cirrhosis and is associated with mortality if untreated. The key components of AIH include hypergammaglobulinaemia, particularly immunoglobulin G (IgG), autoimmune antibodies and interface hepatitis on histology. There are two types of AIH, which are outlined in Table 1. Both types are associated with a number of autoimmune conditions, including coeliac disease and thyroiditis, and their early diagnosis is crucial to prevent complications.

**Table 1:** Comparison of type 1 autoimmune hepatitis and type 2 autoimmune hepatitis.

	AIH-1	AIH-2
Age of onset	Any age but is predominant in adults	Mainly in children
Autoimmune antibodies	ANA and/or anti-SMA	Anti-LKM-1 and/or anti-LC1
Disease course	Variable – can range from indolent to acute Progression to cirrhosis if untreated	Increased propensity to present with ALF Greater risk of rapid progression to cirrhosis

AIH, Autoimmune hepatitis; ALF, acute liver failure; ANA, anti-nuclear antibody; anti-LC1, anti-liver cytosol 1 antibody; anti-LKM-1, anti-liver kidney microsomal 1 antibody; anti-SMA, anti-smooth muscle antibody.

Table content relates to references 4 and 5.

ASC is an overlap syndrome of sclerosing cholangitis (chronic inflammation and progressive fibrosis of the bile ducts) and AIH. ASC is more common in children than adults.^4^ It has a serological profile in keeping with AIH along with evidence of cholangiopathy on imaging, but ALP and GGT are often normal or mildly elevated in early disease. ASC presents with similar symptoms to AIH^5^ but differs in that it affects both sexes equally, whereas AIH is more common in women. ASC also has a greater association with inflammatory bowel disease (IBD), particularly ulcerative colitis, with 45% of children affected compared to 20% of children with AIH.^4^ IBD is rarely associated with AIH-2.^6^ Awareness of this AILD-IBD (including PSC) association is crucial for adult physicians to recognise disease flares and IBD-related complications. ASC-PSC-IBD patients also require regular surveillance colonoscopies throughout adulthood for early detection of colon cancer. Furthermore, many patients with ASC will phenotypically progress to PSC in adulthood, in keeping with the concept of ASC as the ‘inflammatory’ phase of PSC,^7^ highlighting the importance of long-term follow-up and regular reassessment with imaging and, when indicated, liver biopsy. The PSC cohort also require regular monitoring for cholangiocarcinoma.

Treatment of AIH and ASC consists of prednisolone first-line with the addition of azathioprine in cases where steroids are not tolerated or liver function tests do not normalise.^4^ Ursodeoxycholic acid (UDCA) is frequently used in PSC and ASC (where it is given in addition to first-line immunosuppression), although it remains unclear whether it improves outcomes. As with adults, mycophenolate mofetil and more potent immunosuppression can be considered in refractory cases, with ongoing monitoring for treatment complications. Monitoring may include screening for metabolic complications and interval DEXA scans to assess bone density. A key element of management in adult services is assessing the need for ongoing immunosuppression and whether therapy can be safely withdrawn, which is especially relevant for family planning considerations.

AILD typically responds well to immunosuppression, except in cases presenting with ALF, with remission rates of up to 90% for AIH.^4^ LT can be indicated in both AIH and ASC and these patients require long-term monitoring in adult services, particularly due to the risk of AILD recurrence following transplantation. It is also important to be aware of plasma cell-rich rejection hepatitis^8^ in this setting. It is a condition that resembles AIH but develops more commonly in children than adult patients (5–10% vs 1–2% respectively) transplanted for non-AIH aetiologies.^8^ Its treatment is as per the classical AIH pathway.^9^

### Cholestatic liver diseases

#### Progressive familial intrahepatic cholestasis

Progressive familial intrahepatic cholestasis (PFIC) describes a group of rare genetic liver conditions that affect bile acid (BA) pathways, leading to impaired bile flow and BA accumulation in the liver. As BAs accumulate. cholestasis develops. causing inflammation and liver fibrosis, which can lead to end-stage liver failure. PFIC incidence is reported as between 1/50,000 and 1/100,000 births.^10^ The three most common PFIC subtypes are outlined in Table 2.

**Table 2:** Comparison of the three main PFIC subtypes.

	FIC1 deficiency	BSEP deficiency	MDR3 deficiency
Gene mutation	*ATP8B1*	*ABCB11*	*ABCB4*
Protein deficiency	FIC1	BSEP	MDR3
Cholestasis pattern	Low/normal GGT	Low/normal GGT	High GGT and ALP
Biochemical features	Raised ALT/AST, low albumin, elevated BAs	Markedly elevated ALT/AST, elevated BAs	BAs may be normal
Extra-hepatic features	Diarrhoea, pancreatic insufficiency, hearing loss, short stature	No associated extra-hepatic features	No associated extra-hepatic features

ALT, Alanine aminotransferase; ALP, Alkaline phosphatase; AST, Aspartate aminotransferase; BAs, Bile acids; BSEP, Bile Salt Export Pump; FIC1, Familial Intrahepatic Cholestasis Type 1; GGT, Gamma-glutamyl transferase; MDR3, Multi-Drug Resistance protein 3.

Table content relates to references 9, 10 and 14.

Familial intrahepatic cholestasis type 1 (FIC1) commonly presents in the first months of life with jaundice and pruritus. Impaired FIC1 protein leads to a harmful imbalance in bile salt composition. FIC1 is progressive and the majority of these patients undergo LT in childhood.^11^ FIC1’s extra-hepatic manifestations (outlined in Table 2) relate to FIC1 protein expression in multiple tissues. FIC1 is not associated with hepatocellular cancer (HCC).

*ABCB11* mutations impair the bile salt exporter pump (BSEP) protein – the dominant bile acid transporter. BSEP deficiency is the most common PFIC subtype and presents with varying degrees of severity. In adults, milder forms can present with chronic liver disease or intrahepatic cholestasis of pregnancy (ICP).^12^ In infancy, when BSEP deficiency is most common, pruritus and intermittent or progressive jaundice are presenting features. Patients with BSEP deficiency classically have normal/mildly elevated GGT with elevated ALT/AST (typically higher than in FIC1), elevated serum BAs and are more likely to develop portal hypertension than those with FIC1.^12^ Importantly, BSEP deficiency is associated with early HCC and cholangiocarcinoma.^13^ Medical management is limited, with UDCA often ineffective. Apical sodium-dependent bile acid transporter (ASBT) inhibitors, such as odevixibat and maralixibat, can be used to reduce BAs and improve pruritus. Nasobiliary drainage and surgical biliary diversion are effective treatments. LT may be indicated, but symptoms of BSEP deficiency can recur following LT due to development of antibodies that inhibit the normal BSEP protein action.^14^

Multi-drug resistance protein 3 (MDR3) deficiency typically presents with jaundice, pruritus, elevated ALP and elevated GGT either in childhood or adulthood and BAs can be normal.^15^ MDR3 deficiency represents a disease spectrum corresponding to the patient’s *ABCB4* genotype as outlined in Table 3.

**Table 3:** Overview of *ABCB4* variant-associated conditions.

Condition	*ABCB4* variant	Clinical features	Management
MDR3 deficiency	Homozygous or compound heterozygote	Jaundice, pruritus	LT often required in childhood
LPAC	Heterozygous	Cholesterol gallstone formation before age 40 with biliary colic Recurrence of gallstones post-cholecystectomy	UDCA ERC for gallstones Cholecystectomy
ICP	Heterozygous	Pruritus during 2nd or 3rd trimester of pregnancy Can also develop jaundice Raised BAs Symptoms typically resolve soon after delivery Complications including stillbirth	UDCA
DILI	Heterozygous	Jaundice following initiation of drugs including OCPs, antibiotics and psychotropic drugs	Withdrawal of drug UDCA

BAs, Bile acids; DILI, Drug-induced liver injury; ERC, endoscopic retrograde cholangiography; ICP, Intrahepatic cholestasis of pregnancy; LPAC, Low phospholipid-associated cholelithiasis; LT, Liver transplant; MDR3, Multi-Drug Resistance protein 3; OCP, Oral contraceptive pill; UDCA, Ursodeoxycholic acid.

Table content relates to references 14 and 15.

Treatment is often initially with UDCA, but results vary, with a complete response only seen in the mild genotypes. ASBT inhibitors can be effective as second-line options, while LT can be successful in patients who develop end-stage liver disease. Long-term follow up is indicated for patients with *ABCB4* variants, especially if they have abnormal liver function, to monitor for progression to advanced liver disease. There is also an increased risk of HCC and cholangiocarcinoma with particular genotypes.^14^ Annual liver ultrasound and fibroscan is recommended. Genetic testing should be offered to first-degree relatives.^14^

#### Alagille syndrome

Alagille syndrome (ALGS) is an autosomal dominant multi-organ disorder impacting 1/30,000 to 1/50,000 live births.^16^ The large majority of patients have *JAG1* gene mutations, while a small proportion have *NOTCH2* mutations, and in both cases the result is bile duct paucity. Clinical manifestations of ALGS vary in severity and those with milder phenotypes may be diagnosed in adulthood. Liver disease often presents in early life with cholestasis, but recognising which patients will develop end-stage liver disease is challenging. Portal hypertension and cirrhosis occur in 10–50% of patients with hepatic involvement in childhood.^17^ Those with chronic cholestasis can develop severe pruritus and xanthomas. ALGS also consists of extra-hepatic manifestations as outlined in Table 4.

**Table 4:** Extra-hepatic features of Alagille syndrome.

Organ system affected	Key features
Cardiovascular	Pulmonary artery stenosis Tetralogy of Fallot
Renal	Renal dysplasia Renal tubular acidosis
Skeletal	Butterfly vertebrae
Vascular	Intracranial aneurysms
Facial features	Triangular face Prominent forehead Deeply set eyes
Ocular	Posterior embryotoxon Optic disc drusen
Neurological	Moyamoya Intracerebral haemorrhage

Table content relates to reference 17.

Management of hepatic ALGS involves controlling pruritus with conservative measures and medications including UDCA. If pruritus persists, second-line options including colesevelam and rifampicin can be trialled.^18^ ASBT inhibitors can also improve pruritus in ALGS, and maralixibat is licensed for this indication.^18^ If refractory pruritus develops, surgical intervention with partial extra-biliary drainage may be required. LT can be performed for refractory pruritus or end-stage liver disease, and outcomes are positive in both paediatric and adult LT. Only around 10% of patients with ALGS undergo LT as adults.^19^ The extra-hepatic features, particularly the cardiac and renal elements, are important considerations in those undergoing LT, as these can complicate peri-operative haemodynamics and postoperative immunosuppression management respectively. LT does not resolve the extra-hepatic features of ALGS. Fat-soluble vitamin replacement is recommended. These may be deficient secondary to cholestasis, and replacement prevents bone fractures and visual impairment. HCC in ALGS is rare, but can occur without cirrhosis.^20^ In patients with cirrhosis 6-monthly ultrasound and AFP testing should be undertaken, while in those without cirrhosis annual ultrasound and AFP levels are appropriate.

## Funding

This research did not receive any specific grant from funding agencies in the public, commercial, or not-for-profit sectors.

## CRediT authorship contribution statement

**Vikram Bains:** Writing – original draft, Conceptualization. **Deepak Joshi:** Writing – review & editing, Conceptualization.

## Declaration of competing interest

The authors declare that they have no known competing financial interests or personal relationships that could have appeared to influence the work reported in this paper.
